# Quantitative assessment of Public Health and Social Measures implementation and relaxation on influenza transmission during COVID-19 in China: SEIABR and GBDT models

**DOI:** 10.7189/jogh.14.05038

**Published:** 2024-12-27

**Authors:** Yuxi He, Kaiwei Luo, Han Ni, Wentao Kuang, Liuyi Fu, Shanghui Yi, Yuan Lv, Wenting Zha

**Affiliations:** 1 Hunan Key Laboratory of Molecular Epidemiology, School of Medicine, Hunan Normal University, Changsha, Hu Nan, China; 2 Hunan Provincial Center for Disease Control and Prevention (Hunan Academy of Preventive Medicine), Changsha, Hu Nan, China; 3 Hunan Workstation for Emerging infectious Disease Control and Prevention, Changsha, Hu Nan, China

## Abstract

**Background:**

Since 2019, China has implemented Public Health and Social Measures (PHSMs) to manage the coronavirus disease 2019 (COVID-19) outbreak. As the threat from SARS-CoV-2 diminished, these measures were relaxed, leading to increased respiratory infections and strained health care resources by mid-2023.

**Methods:**

The study utilised WHO's FluNet and Oxford's COVID-19 Government Response Tracker to assess how policy shifts have affected influenza. It examined changes in influenza incidence, subtype prevalence, and epidemic cycles over three periods: pre-COVID-19 and pre-PHSMs, during COVID-19 and PHSMs, and post-COVID-19 and post-PHSMs. The SEIABR model estimated the transmission probability () and real-time reproduction number () across these periods, while a gradient boosting decision tree (GBDT) analysed the effects of PHSM indicators on influenza transmission.

**Results:**

Results indicate that before PHSMs, the average incidence was 4.87 per 100 000, with a β-value of (7.95 ± 1.27) × 10^−10^ and *R_t_*-value of 1.21 ± 0.16. During PHSMs, incidence dropped to 2.55 per 100 000, and β decreased to (3.17 ± 0.75) × 10^−10^ (*R_t_*-value of 0.86 ± 0.20). Post-PHSMs, the incidence surged to 17.00 per 100 000, with β rising to 8.36 × 10^−10^ (*R_t_*-value of 2.25). The GBDT model identified testing policies, public information campaigns, and workplace closures as the most impactful PHSM indicators.

**Conclusions:**

PHSMs effectively mitigated the spread of influenza, providing a foundation for future policy development to prevent respiratory diseases.

Influenza, commonly known as the flu, is an acute viral respiratory infection caused by influenza viruses that affects the respiratory tract. It poses a risk to all demographic groups but particularly impacts children and the older people, who often have lower immunity levels [1]. The main types of influenza viruses, A H1N1, H3N2 (each influenza A subtype is characterised by numbering both the HA and NA proteins), and B Victoria/Yamagata, are known as seasonal influenza viruses and have been responsible for several global pandemics [2]. Those infected with influenza experience mild symptoms such as fever, headache, dry cough, weakness, and sore throat following an incubation period. However, a small percentage may develop more severe complications like viral or secondary pneumonia, which can lead to multi-organ failure or even death in extreme cases [3,4]. In the latter half of 2023, mainland China reported a peak of 13 799 influenza cases, indicating a prolonged and widespread epidemic. During this period, there was notable co-prevalence and co-infection with multiple pathogens, including influenza A and B viruses, parainfluenza viruses, respiratory syncytial viruses (RSV), and SARS-CoV-2. The simultaneous circulation of these pathogens can place a significant strain on health care resources, potentially exceeding the capacity typically challenged by a single-pathogen outbreak.

COVID-19, caused by the SARS-CoV-2 virus, was first identified in Wuhan, China, in December 2019 and rapidly spread globally within months [5]. On 20 January 2020, China classified COVID-19 as a ‘Category B infectious disease’ but applied ‘Category A’ measures to manage it. These unique PHSMs involved quarantining patients, designating outbreak zones as high or low-risk, and conducting widespread nucleic acid testing. As of 8 January 2023, the National Health Commission (NHC) of China downgraded the management of COVID-19 to ‘Category B with measures for Category B,’ leading to the cessation of previously stringent PHSMs such as isolation of infected individuals and closure of specific areas. The implementation and subsequent relaxation of PHSMs have significantly altered lifestyles and health statuses across populations. Researchers like Zeng [6], Chan [7], and Olsen [8] have explored the epidemiological characteristics of seasonal influenza in PHSMs in different countries using influenza surveillance data. Zhang’s study on the 2022–2023 influenza season in Beijing compared to the 2014–2020 seasons highlighted changes in epidemic occurrence, intensity, duration, and transmissibility of influenza during the COVID-19 era [9]. The control of influenza involves three key elements. First, controlling the transmission source entails the early identification and management of infected individuals, thereby reducing virus spread through timely diagnosis and treatment. Second, the critical strategy for interrupting transmission pathways lies in implementing PHSMs, which can effectively halt the spread of the virus within populations, particularly during outbreaks [10,11]. Finally, strategies to protect susceptible populations, especially targeting the child, elderly and individuals with chronic illnesses, can significantly reduce their risk of infection [12].

These studies collectively indicate that PHSMs targeting COVID-19 have had some impact on the characteristics of influenza incidence. However, comprehensive reports systematically analysing the changes in seasonal influenza incidence, dominant subtypes, epidemic cycles, and transmissibility before, during, and after the COVID-19 epidemics are scarce. Moreover, detailed studies quantifying the impact of different PHSM policies on influenza incidence are rarer, indicating a significant gap in current research.

Mathematical modelling plays a vital role in developing and evaluating strategies aimed at preventing and controlling influenza. This study undertook a comprehensive analysis by calculating and comparing influenza incidence rates across three distinct periods: the pre-COVID-19 and pre-PHSMs period (2010–2019), the COVID-19 and PHSMs period (2020–2022), and the post-COVID-19 and post-PHSMs period (2023). Utilising the SEIABR model, the research explored changes in influenza incidence, epidemic cycles, and transmission capacity over an extended timeframe from 2010 to 2023 within mainland China (excluding Hong Kong). Building on the SEIABR model's results, the study further analysed the effect of specific PHSMs during the COVID-19 period using a Gradient Boosting Decision Tree (GBDT) model, which quantified the relative importance of each measure and identified the most effective strategies for controlling influenza transmission. Overall, this research provides valuable insights to inform future influenza prevention and control strategies.

## METHODS

Below are the data used in this paper and the research methods employed.

### Data source

This study collected demographic data from the National Bureau of Statistics of China and influenza case data from the WHO’s FluNet (2010–2023). The PHSM data from the Oxford COVID-19 Government Response Tracker (2020–2022) was analysed by converting daily records into weekly averages and assigning intensity scores for impact assessment.

#### Demographic data

The study sourced annual demographic data, including the end-of-year total population, natural birth rate, and natural death rate for China from 2010 to 2023, from the official website of the National Bureau of Statistics of China (http://www.stats.gov.cn). This data provided a foundational context for assessing the broader impacts of influenza on the population over the study period.

#### Data on reported cases of influenza

We collected influenza case data for China from 2010 to 2023 from the FluNet section of the WHO official website (https://www.who.int/tools/flunet). This comprehensive data set contains weekly totals of individuals tested, cases of influenza-like illness (ILI), distinguishing between influenza-positive and influenza-negative patients, and the various subtypes of influenza. We segmented the influenza data into three distinct periods according to the nodes of the COVID-19 pandemic and the implementation of PHSMs:

1. Pre-COVID-19 and pre-PHSMs period (2010–2019). During this timeframe, COVID-19 had not yet emerged. This period establishes a baseline that helps us understand the dynamics of influenza transmission and incidence under usual conditions, unaffected by COVID-19-related PHSMs. This comparison is crucial for gauging the direct impact of such measures on influenza patterns.

2. COVID-19 and PHSMs period (2020–2022). In this phase, COVID-19 was recognised as a ‘category B infectious disease’ but treated with the stringent measures typically reserved for a ‘category A disease’. These measures included the isolation of infected individuals, the designation of specific areas as high-risk zones, and extensive nucleic acid testing, among other interventions. This period allows for the analysis of influenza data under PHSMs.

3. Post-COVID-19 and post-PHSMs period (2023). By this time, COVID-19 management had been downgraded to ‘category B with measures for Category B’, leading to the cessation of most stringent PHSMs such as isolation, area blockades, and sanitary quarantines of transport. This recent period provides insights into influenza patterns following the relaxation of PHSMs.

To address any missing data within these defined periods, we obtained supplementary information by accessing the weekly influenza case reports from the influenza surveillance network of the National Influenza Center of China (http://www.chinaivdc.cn/cnic), which ensures a comprehensive data set for analysing the impact of varying public health strategies on influenza trends across different periods.

#### PHSMs data

This study utilised data from the COVID-19 Government Response Tracker (OxCGRT) (https://www.bsg.ox.ac.uk/research/covid-19-government-response-tracker), developed by the University of Oxford. This resource tracks and quantifies government responses to the COVID-19 pandemic across 165 countries, providing a detailed view of the strictness and scope of various PHSMs. For this research, we have selected specific PHSMs that are hypothesised to influence influenza transmission, based on prior studies. These measures include containment and closure policies (C) and health system policies (H), as classified by the OxCGRT, reflecting key government strategies to mitigate virus transmission and bolster public health responses: C1 (school closing), C2 (workplace closing), C3 (cancel public events), C4 (restrictions on gatherings), C5 (close public transport), C6 (stay at home requirements), C7 (restrictions on internal movement), C8 (international travel controls), H1 (public information campaigns), H2 (testing policy), H3 (contact tracing), H6 (facial coverings).

In this paper, we selected data spanning from 1 January 2020 to 31 December 2022. Then, we converted the raw daily data into weekly averages to align with the influenza data collection intervals, and initially categorised the PHSMs into five levels (0–4), with level 0 indicating no measures and levels 1–4 indicating increasing strictness. For analytical simplicity, levels 1 to 4 were further condensed into two categories based on the strictness and scope of implementation within the country. Each level was then assigned an intensity score out of 100, facilitating a quantifiable analysis of the impact of these measures on influenza transmission. The appendix in the **Online Supplementary Document** provides details on the scoring methodology and categorisation.

### Transmission dynamic model

In this study, we have developed the SEIABR model to analyse the transmission dynamics of influenza. This model categorises the population into six distinct compartments, which are: susceptible (S), incubating (E), infectious (I), asymptomatic (A), non-visit (B), and removed (R). Given that influenza often presents mild symptoms or remains asymptomatic in many cases, a significant portion of infected individuals may self-medicate and avoid formal medical care, leading to under-reporting. To address this, the SEIABR model expands on the basic SIR model by incorporating E, A, and B compartments. These additions allow for a more detailed simulation of infection and transmission pathways, making the model particularly suitable for diseases with asymptomatic spread and unreported cases.

**Figure 1:**
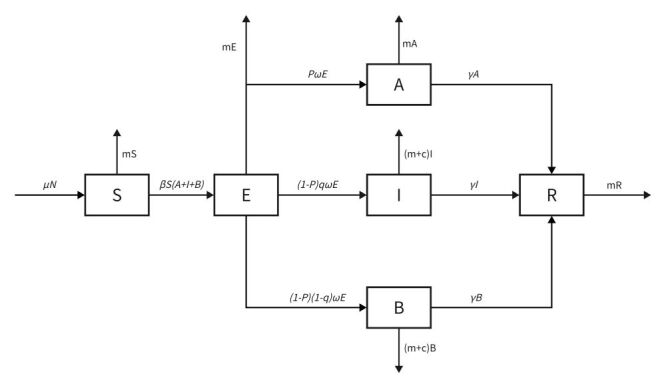
Schematic diagram of the model flow.

**Figure 1** presents the flow diagrams, **Table 1** provides a description of the compartments/variables, and **Table 2** outlines the parameters, including β (rate of infection of susceptible individuals by exposed individuals), *p* (fraction of exposed individuals who remain asymptomatic), *q* (visit ratio of symptomatic individuals), among others.

**Table 1:** Description of variables in the model

Variables	Meaning
S	Number of susceptible human population
E	Number of exposed human population
I	Number of symptomatic human population
A	Number of asymptomatic human population
B	Number of non-visit population
R	Number of recovered human population
N	Number of total population

**Table 2:** The meaning and value of each parameter in the SEIABR model*

Parameters	Meaning	Units	Value
β	Rate of infection of susceptible with exposed human	person^−1^day^−1^	Assumed
*p*	Fraction of exposed individuals not showing clinical symptoms	–	1/3 [13,14]
ω	Rate of incubation	person^−1^day^−1^	1/7–1/2 [15,16]
*q*	Visit ratio of symptomatic individuals	–	0.25–0.70 [17]
γ	Recovery rate	person^−1^day^−1^	1/7 [18,19]
μ	Natural birth rate	day^−1^	Related data
*m*	Natural mortality rate	day^−1^	Related data
*v*	The average influenza vaccination rate	–	0.03–0.10 [20]
*c*	Mortality of influenza		0.003 [21]

*The SEIABR model includes six compartments: susceptible (S), incubating (E), infectious (I), asymptomatic (A), non-visit (B), and removed (R).

There are several assumptions of the SEIABR model:

1. A susceptible person can be infected by an infected person and become newly infected. The rate of infection of a susceptible person by an infected person is β.

2. A susceptible person may become symptomatic or asymptomatic when infected. The probability of one of these becoming asymptomatically latently infected is *p*.

3. The probability that a susceptible person will be infected and seek medical attention is *q*.

4. The recovery rate of symptomatic patients, asymptomatic patients, and non-visit patients is .

5. Both infected and visit patients and infected non-visit patients can die from influenza virus infection with a case fatality rate of *c*.

6. The recovering individual will not be re-infected by the infected individual and will not infect others.

Assuming *S* (*t*) *= N* (*t*) (1*−v*), the model is represented by a system of differential equations:

*dS*/*dt* = *μN* − *βSE* − *mS*

*dE*/*dt* = *βSE − mE − ωE*

*dI*/*dt* = (*1 − p*) *qωE −* (*m + c*) *I − γI*

*dA*/*dt = pωE − mA − γA*

*dB/dt =* (*1−p*) (*1−q*) *ωE −* (*m + c*) *B − γB*

*dR/dt =* (*A + I + B*) *γ − mR*

### Real-time regeneration number calculation

The time-varying reproduction number, denoted as *R_t_*_,_ is a critical epidemiological metric that quantifies the potential for transmission of an infectious disease within a population under specific conditions at a given time. It describes the average number of secondary infections produced by a single infectious individual in a population where some individuals are no longer susceptible due to previous infections or vaccinations.

When *R_t_*>1, The disease will likely spread more extensively within the population, which indicates each infected individual, on average, transmits the disease to more than one other person, leading to a potential increase in cases and a higher risk of an outbreak.

When *R_t_* = 1, The disease maintains a steady state where each infected individual, on average, transmits the disease to another person. This scenario is referred to as the ‘disease-free equilibrium’, where the number of new cases stabilises, but the disease remains present in the population.

When *R_t_*<1, The spread of the disease is likely to decline, leading to a decrease in case numbers over time. If sustained, this can result in the eventual elimination of the disease from the population, as new infections are not sufficient to maintain the outbreak [22].

The effective reproduction number, *R_t_*, is calculated using the next-generation matrix method, which captures how new infections arise from existing cases in a population.

1. Next-generation matrix. Two matrices are defined, *F* (representing new infections) and *V* (representing transitions between compartments).

2. Simplified matrices. The matrix *F* includes the transmission rate, β, and the number of susceptible individuals, S. The matrix *V* includes various parameters representing recovery rates and losses from the system.

3. Effective reproduction number. The next-generation matrix is used to derive *R_t_* through the spectral radius of the matrix *FV ^−1^*. Thus, the formula for *R_t_* can be expressed as:

*R_t_* = *βS* / (*m + ω*)

### GBDT model

The Gradient Boosting Decision Tree (GBDT) model is a robust machine learning algorithm derived from ensemble learning. It is particularly effective at handling large data sets, accommodating missing data, managing diverse input variables, and avoiding the need for strict assumptions. These qualities align well with the PHSM data sets used in this study, allowing GBDT to effectively adapt to the data’s varying characteristics. Additionally, GBDT’s capability to rank feature importance is invaluable in identifying which interventions, such as social distancing or travel restrictions, exert the greatest influence on disease control – a novel aspect of the research.

The GBDT model was trained using labelled data, with the PHSMs features serving as input variables and the reported influenza case numbers (Cases) as the output variable, while assuming that external confounders – such as population density, health care infrastructure, or weather patterns – uniformly affect the impact of each PHSM across the regions considered. To further clarify, the GBDT algorithm follows these key steps:

Input training set with maximum number of iterations T, loss function L and output intense learner f(x).

1. Initialisation: obtain a tree with only a root node, where the output value of the root node minimises L:



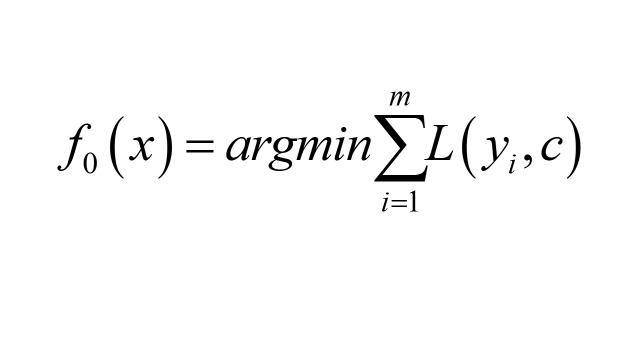



2. Compute the negative gradient of the t(1-T) round for the i(1 ~ m) sample:



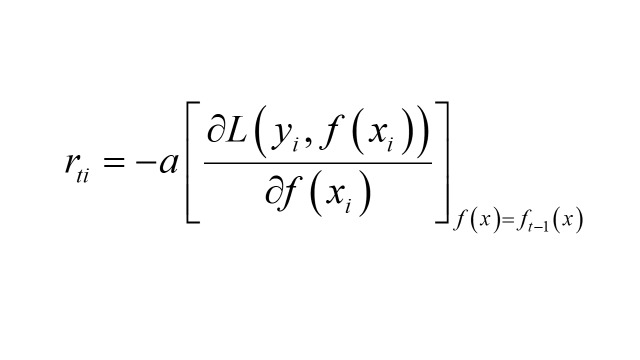



2. Fit a CART regression tree to rt,i, obtaining the leaf node region Rt,j for the mth tree, and calculate the output value for each region:



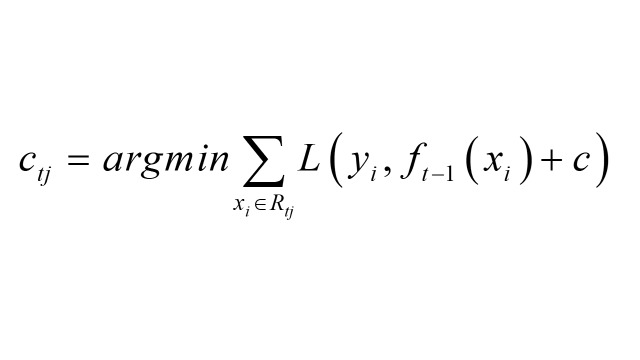



3. Update and obtain the final regression tree:



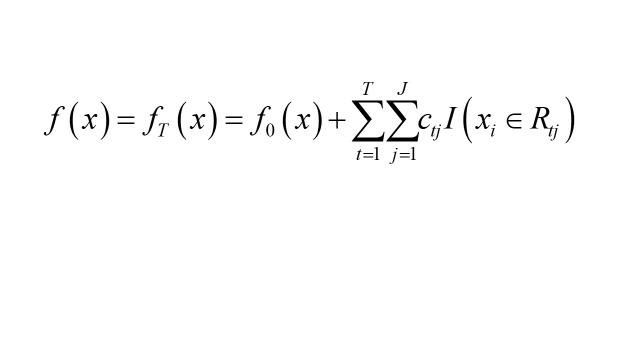



### Statistical software

We used Berkeley Madonna 10.5.1 (Berkeley Madonna Inc., University of California, Berkeley, USA, 2020) software for building and fitting the SEIABR model, Matlab R2023a (MathWorks, Natick, MA, USA, 2023) for deriving and computing *R_t_* formulae, SPSS 25 (IBM Corp., Armonk, NY, USA, 2017) for Spearman's correlation analysis, Python 3.12 (Python Software Foundation, Wilmington, DE, USA, 2023) for GBDT modelling, and Origin 2022 (OriginLab, Northampton, MA, USA, 2022) for data visualisation.

## RESULTS

In this section, we present the implementation and relaxation of PHSMs, their impact on influenza incidence, subtype composition, epidemic cycles, transmission capacity, and the importance of specific PHSM indicators.

### Implementation of PHSMs

**Figure 2:**
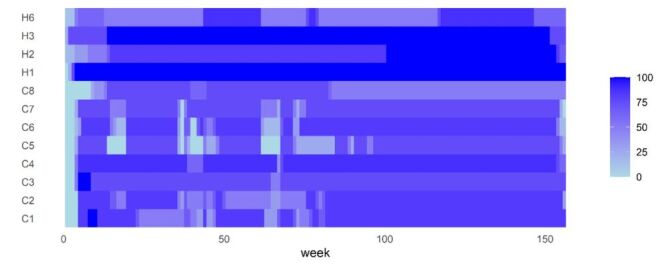
Temporal Heatmap of Public Health and Social Measures.

A brief descriptive analysis of the basic PHSMs in the COVID-19 & PHSMs period (2020–2022, 156 weeks total). There was no change in measures C3 (cancel public events), H1 (public information campaigns), and H3 (contact tracing), there was slight fluctuation in the changes in the measures H2 (testing policy), C4 (restrictions on gatherings), and C8 (international travel controls), and there were significant changes in the measures C1 (school closing), C2 (workplace closing), C5 (close public transport), C6 (stay at home requirements), C7 (restrictions on internal movement), and H6 (facial coverings) (**Figure 2**).

### Impact of PHSMs on influenza incidence

Changes in influenza incidence rates, subtype composition, and epidemic cycles across different periods are examined, with the detailed findings summarised below.

#### Impact of PHSMs on the influenza incidence rate

We have calculated the annual influenza incidence rate by dividing the number of reported influenza cases by the total population, the specific values are presented in **Table 3** and **Table 4**. Here are the observed trends across different periods:

**Table 3:** China’s demographic statistics

Year	N (million)	μ* (%)	*m*† (%)
2010	134 091	1.19	0.71
2011	134 735	1.19	0.71
2012	135 404	1.21	0.72
2013	136 272	1.21	0.72
2014	136 782	1.24	0.72
2015	137 462	1.21	0.71
2016	138 271	1.30	0.71
2017	139 008	1.24	0.71
2018	139 538	1.09	0.71
2019	140 005	1.05	0.71
2020	141 178	0.85	0.71
2021	141 260	0.75	0.72
2022	141 175	0.68	0.74
2023	140 967	0.64	0.79

*μ represents the natural birth rate.

†*m* represents the natural mortality rate.

**Table 4:** Annual incidence of influenza in China, 2010–2023

Period	Year	Total population (× 10^4^)	Cases	Incidence (/10^5^)
Pre-COVID-19 and pre-PHSMs period	2010	134 091	44 166	3.29
	2011	134 735	21 515	1.60
	2012	135 404	44 371	3.28
	2013	136 072	32 992	2.42
	2014	136 782	71 641	5.24
	2015	137 462	70 219	5.11
	2016	138 271	87 672	6.34
	2017	139 008	99 038	7.12
	2018	139 538	80 296	5.75
	2019	140 005	119 783	8.56
During the COVID-19 and PHSMs period	2020	141 178	25 514	1.81
	2021	141 260	26 068	1.85
	2022	141 175	56 339	3.99
Post-COVID-19 and post-PHSMs period	2023	140 967	239 595	17.00

COVID-19 – coronavirus disease 2019, PHSMs – Public Health and Social Measures

Pre-COVID-19 and pre-PHSMs period (2010–2019). The mean influenza incidence rate was 4.87 per 100 000. The lowest incidence rate was observed in 2011 (1.60 per 100 000) and the highest in 2019 (8.56 per 100 000), indicating a generally increasing trend.

COVID-19 and PHSMs period (2020–2022). During this period, the mean incidence rate dropped to 2.55 per 100 000. The year 2020 saw the lowest rate at 1.81 per 100 000, and 2022 the highest at 3.99 per 100 000. These figures are significantly lower than those observed in the pre-COVID-19 and pre-PHSMs period, reflecting the impact of stringent PHSMs.

Post-COVID-19 and post-PHSMs period (2023). After the PHSMs were terminated, the influenza incidence rate surged to 17.00 per 100 000, which is 3.5 times higher than the average rate during the pre-COVID-19 and pre-PHSM period and 6.7 times higher than the average during the COVID-19 pandemic.

#### Impact of PHSMs on the influenza subtype composition

**Figure 3:**
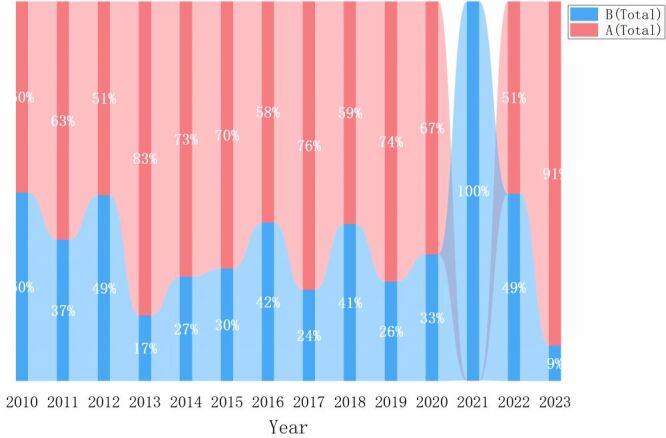
Percentage of influenza subtypes.

The subtype composition of influenza, primarily types A and B, offers insight into the dominant strains circulating within the population. The proportion of these subtypes is calculated by dividing the number of cases of each subtype by the total number of influenza cases. The specific values are presented in **Table 5** and A and B subtype influenza proportions are visualised in **Figure 3**. Here is an analysis of how these proportions have shifted across different periods:

**Table 5:** Number and composition of different types of influenza cases in China, 2010–2023

Period	Year	Different types of influenza		Total number of cases
		**Type A**	**Type B**	
Pre-COVID-19 and pre-PHSMs period	2010	22 233 (50.34)	21 933 (49.66)	44 166
	2011	13 520 (62.84)	7995 (37.16)	21 515
	2012	22 671 (51.09)	21 700 (48.91)	44 371
	2013	27 295 (82.73)	5697 (17.27)	32 992
	2014	51 943 (72.50)	19 698 (27.50)	71 641
	2015	49 456 (70.43)	20 763 (29.57)	70 219
	2016	51 151 (58.34)	36 521 (41.66)	87 672
	2017	75 241 (75.97)	23 797 (24.03)	99 038
	2018	47 109 (58.67)	33 187 (41.33)	80 296
	2019	88 423 (73.82)	31 360 (26.18)	119 783
During the COVID-19 and PHSMs period	2020	17 015(66.69)	8499(33.31)	25 514
	2021	35 (0.13)	26 033(99.87)	26 068
	2022	28 571 (50.71)	27 768(49.29)	56 339
Post-COVID-19 and post-PHSMs period	2023	217 463 (90.76)	22 132(9.24)	239 595
Total		712 126 (69.87)	307 083(30.13)	1 019 209

COVID-19 – coronavirus disease 2019, PHSMs – Public Health and Social Measures

Pre-COVID-19 and pre-PHSMs period. On average, influenza A constituted 65.67% of cases, nearly twice the proportion of influenza B at 34.33%. In specific years like 2010, 2012, 2016, and 2018, the proportions of influenza A and B were roughly equal. In other years, influenza A predominated over influenza B.

COVID-19 and PHSMs period. In 2020, the distribution maintained a similar pattern to previous years, with influenza A accounting for twice as many cases as influenza B. However, in 2021, there was a significant shift, with influenza B dominating at 99.87%, becoming the major component of the disease. By 2022, the proportions of subtypes A and B had nearly equalised once again.

Post-COVID-19 and post-PHSMs period. Following the termination of PHSMs, influenza A surged to account for 90.76% of cases, a proportion higher than at any time in previous years.

#### Impact of PHSMs on the epidemic cycle of influenza

**Figure 4:**
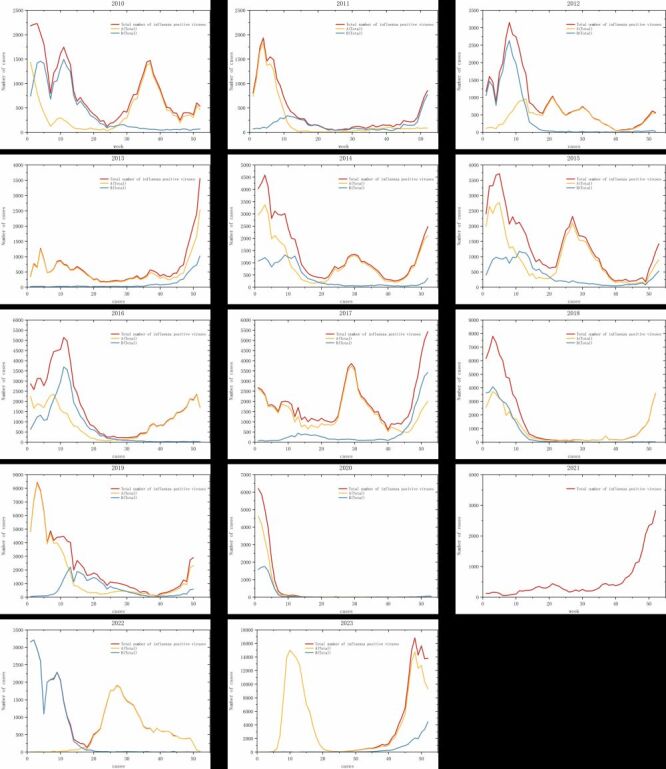
Time series chart of 2010 ~ 2023.

To analyse the impact of PHSMs on the influenza epidemic cycle in China, we visualised and plotted data on reported influenza cases as a time series (**Figure 4**). This approach allowed us to observe how the epidemic cycles varied across different periods.

In pre-COVID and pre-PHSMs period, typically, there were two to three influenza peaks per year, with major seasonal outbreaks occurring at the beginning or end of the year (winter and spring). And we often observed minor peaks during the mid-year (summer). The duration of these outbreaks ranged from 10 to 15 weeks, with a general upward trend in peak case numbers, culminating in over 8000 cases in 2019.

COVID-19 and PHSMs period. During this period, characterised by stringent public health measures, we observed a notable reduction in the frequency of influenza peaks, with only one significant peak per year in both 2020 and 2021. These peaks still occurred towards the beginning and end of the year, respectively. Additionally, the magnitude of these peaks was significantly lower, with the lowest recorded at 2820 cases in 2021.

Post-COVID-19 and post-PHSMs period: after the termination of PHSMs, the influenza pattern reverted to pre-COVID-19 and pre-PHSM conditions with multiple peaks throughout the year. However, there was a dramatic increase in cases, reaching a peak incidence of 16 816 cases in week 48 of 2023.

### Impact of PHSMs on the transmission capacity of influenza

**Figure 5:**
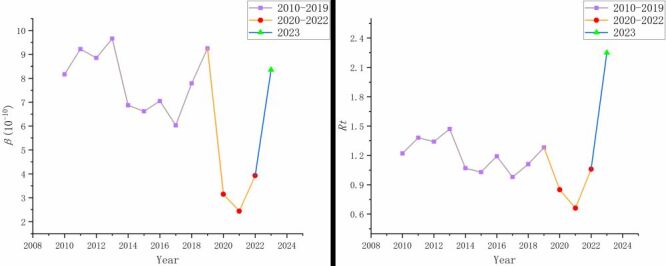
Point line diagram of the *β*-value and the *Rt*-value.

The transmission capacity of influenza, represented by the β-value and *R_t_*-value, has been analysed from 2010 to 2023 using model fitting and computation, where higher values signify a greater potential for transmission. The specific values are presented in **Table 6**, and the visual representation is provided in **Figure 5**. Here's a breakdown of these values across different periods:

**Table 6:** The β-value and *R_t_*-value in China, 2010–2023

Period	Year	β* (× 10^−10^)	Increment (grand total)	Increment (per year)	*R_t_*†	Increment (grand total)	Increment (per year)
Pre-COVID-19 and pre-PHSMs period	2010	8.17	–	–	1.22	–	–
	2011	9.22	1.05	1.05	1.38	0.16	0.16
	2012	8.85	0.68	−0.37	1.34	0.12	−0.04
	2013	9.66	1.49	0.81	1.47	0.27	0.13
	2014	6.87	−1.3	−2.79	1.07	−0.15	−0.40
	2015	6.62	−1.55	−0.25	1.03	−0.19	−0.05
	2016	7.05	−1.12	0.43	1.19	−0.03	0.16
	2017	6.03	−2.14	−1.02	0.98	−0.24	−0.21
	2018	7.79	−0.38	1.76	1.11	−0.11	0.13
	2019	9.25	1.08	1.46	1.28	0.06	0.17
During the COVID-19 and PHSMs period	2020	3.15	−5.02	−6.10	0.85	−0.37	−0.43
	2021	2.44	−5.73	−0.71	0.66	−0.56	−0.19
	2022	3.93	−4.24	1.49	1.06	−0.16	0.40
Post-COVID-19 and post-PHSMs period	2023	8.36	0.19	4.43	2.25	1.03	1.19

*β represents the rate of infection of susceptible individuals by exposed individuals.

†*R_t_* denotes the effective reproduction number, which reflects how new infections arise from existing cases within a population.

Pre-COVID-19 and pre-PHSMs period: the average β-value was (7.95 ± 1.27) × 10^−10^, with the lowest β-value recorded at (6.03 × 10^−10^) in 2017 and the highest at (9.25 × 10^−10^) in 2019, exhibited relatively small fluctuations. The average *R_t_*-value was 1.21 ± 0.16, ranging from a low of 0.98 in 2017 to a high of 1.47 in 2013. These values indicate fluctuations around the critical threshold of 1.

COVID-19 and PHSMs period: there was a notable reduction in the β-value, with an average of (3.17 ± 0.75) × 10^−10^. The average *R_t_*-value was 0.86 ± 0.20, which is significantly below 1. The β-value and *R_t_*-value indicate effective suppression of influenza transmission during this period.

Post-COVID-19 and post-PHSMs period: following the termination of PHSMs, there was a significant increase in the β-value to (8.36 × 10^−10^), aligning closely with pre-pandemic levels. And the *R_t_*-value surged to 2.25, significantly higher than during the pandemic and the pre-pandemic period.

### Importance of the impact of different PHSMs on influenza

**Figure 6:**
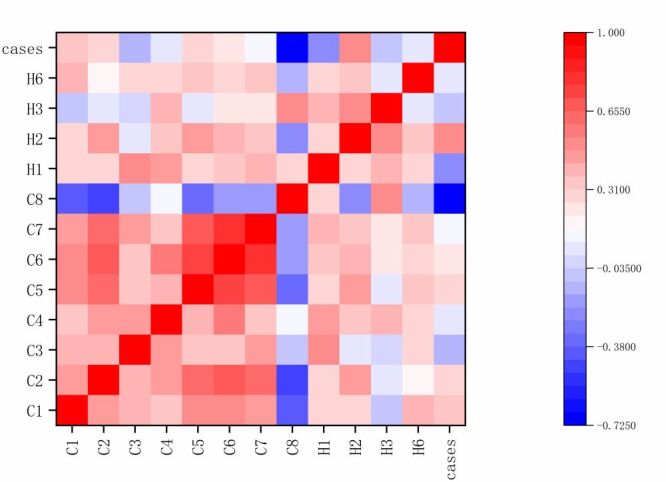
Heatmap of Public Health and Social Measures-influenza cases.

The analysis utilised Spearman's correlation to assess the relationship between the number of reported influenza cases and various PHSMs implemented during the study period. The findings indicate significant correlations between the reported cases of influenza and seven specific measures: C1 (school closing), C2 (workplace closing), C5 (close public transport), C6 (stay at home requirements), C8 (international travel controls), H1 (public information campaigns) and H2 (testing policy) (**Figure 6**), whereas the specific values of the PHSMs indicators are presented in **Table 7**. Among these, C8 (international travel controls) showed the strongest negative correlation with the number of reported influenza cases, with a correlation coefficient of *r* = −0.678 and a significance level of *P* < 0.001.

**Table 7:** Spearman Correlation coefficients between PHSMs indicators and reported influenza cases

PHSMs indicators	r*	*P-*value
C1	0.313	0.000†
C2	0.293	0.000†
C3	–0.145	0.070
C4	0.023	0.780
C5	0.273	0.001†
C6	0.228	0.004†
C7	0.093	0.246
C8	–0.678	0.000†
H1	–0.238	0.003†
H2	0.531	0.000†
H3	–0.044	0.587
H6	0.043	0.594

PHSMs – Public Health and Social Measures

*r represents the correlation coefficient.

†Denotes statistical significance at τ<0.05.

**Figure 7:**
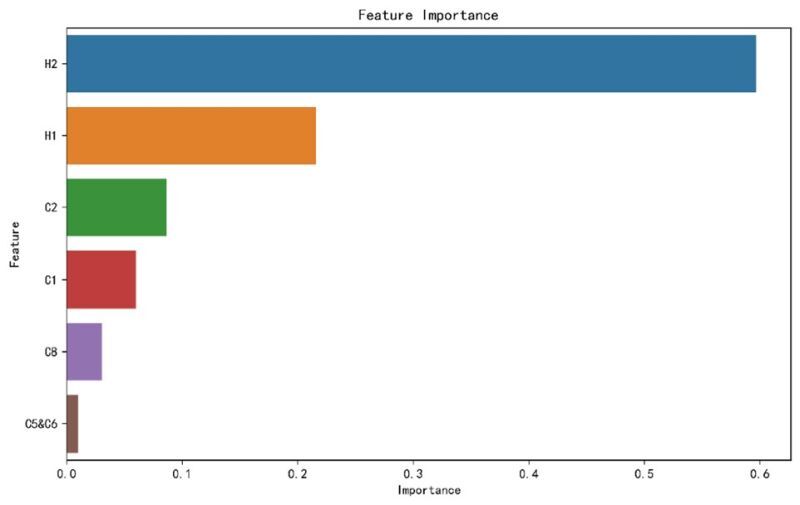
Bar chart of feature importance in gradient boosting decision tree.

After conducting a correlation analysis, the study moved forward with an important analysis of the PHSMs. Due to the strong correlation between C5 (close public transport) and C6 (stay at home requirements), these indicators were amalgamated into a single measure to simplify the model and enhance its interpretability. An optimisation process for parameters was integrated into the GBDT algorithm, utilising grid search to find the optimal parameter combination. The search process involved 5-fold cross-validation, with a learning rate of 0.05, a maximum tree depth of 3, and 300 iterations. We depict the bar chart (**Figure 7**) of feature importance below, showing that the top three PHSM indicators influencing the number of reported influenza cases are H2 (testing policy), H1 (public information campaigns), and C2 (workplace closing).

## DISCUSSION

Exploring the relationship between COVID-19 and influenza epidemics is a pertinent issue in epidemiology and infectious disease research. Although caused by different pathogens, both diseases are respiratory infections and share similarities in transmission routes. This paper statistically analyses influenza incidence data and constructs models to quantitatively assess the changes in influenza incidence, epidemic cycles, and transmission capacity over different periods, focusing on the emergence of COVID-19 and the implementation of PHSMs which are specifically designed to control COVID-19. Additionally, this analysis examines how different categories and intensities of PHSMs implementation affect influenza incidence, offering valuable insights for developing policies aimed at preventing and controlling influenza and other respiratory infections.

The response to COVID-19 has notably enhanced China's capabilities in sentinel surveillance, risk assessment, and emergency preparedness and response [23]. During the pandemic, measures such as C3 (cancel public events), C4 (restrictions on gatherings), C8 (international travel controls), H1 (public information campaigns), H2 (testing policy), and H3 (contact tracing) exhibited minimal changes. However, other measures such as C1 (school closing), C2 (workplace closing), C5 (close public transport), C6 (stay at home requirements), C7 (restrictions on internal movement), and H6 (facial coverings) were significantly more dynamic. These measures were initially implemented in 2020 and subsequently adjusted to respond to changes in the pandemic situation. Overall, their scope of control expanded accordingly. The intensity of PHSMs peaked in 2021 and remained high until the complete cessation at the end of 2022.

In seasonal influenza epidemics in China, the predominant strains typically include type A (H1N1) pdm09, H1N1, H3N2, and type B Victoria, and Yamagata subtypes [24-26]. In the pre-COVID-19 & pre-PHSMs period, influenza A consistently dominated over influenza B, except in 2010, 2012, 2016, and 2018, when the ratio of influenza A to B cases was approximately equal. During the COVID-19 & PHSMs period, the proportion of influenza A was twice that of influenza B in 2020. In 2021, however, influenza B viruses accounted for the vast majority of cases (99.87%), with influenza A cases dropping to just 0.13%, consistent with the overall findings of contemporaneous studies in China [27-29]. In 2022, the proportions of influenza A and B equalized once again. With the comprehensive relaxation of PHSMs, influenza A has re-emerged as the predominant circulating virus, accounting for 90.76% of cases in 2023. This fluctuation raises questions about a potential competitive interaction between influenza A and B viruses and SARS-CoV-2 during the pandemic, which may have influenced the transmissibility dynamics of influenza A. Further research is needed to explore these dynamics and their implications for influenza epidemiology [30].

**Figure 8:**
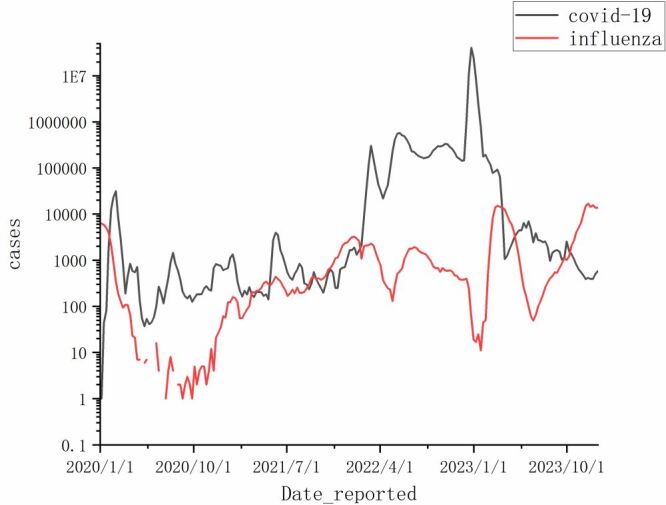
Semilogarithmic line graph of reported influenza and coronavirus disease 2019 cases.

Regarding the epidemic cycle, before COVID-19, influenza epidemics typically exhibited two to three peaks annually, primarily during the winter and spring seasons spanning weeks 45 to 15, aligning with the findings of international studies [31-33]. Additionally, some years displayed a minor peak between weeks 25 to 35 (mid-year). During the COVID-19 & PHSMs period, the frequency of influenza peaks reduced, occurring only once annually, with peaks observed at the beginning (weeks one to four) and the end of the year (weeks 46 to 52), respectively. The mid-year minor peak was absent, likely due to the reduced summer travel influenced by public health restrictions. According to the China Tourism Research Institute, travel restrictions significantly decreased summer outings, with the total travel during the COVID-19 epidemic dropping to an average of 3.07 billion from 6.01 billion in 2019. To analyse the relative prevalence trends of influenza and COVID-19, we created a semi-logarithmic time series plot, which used time as the horizontal coordinate and the cases as the vertical coordinate. We observed that, except for the period from January 2021 to February 2022, when influenza and SARS-CoV-2 were co-prevalent, these pathogens exhibited alternating dominance. Specifically, during peaks of the COVID-19 pandemic, influenza incidence reports were lower, and conversely, when influenza peaks were high, SARS-CoV-2 reports were lower (**Figure 8**). This phenomenon, referred to by Wang et al. as the ‘see-saw effect’, demonstrates a negative correlation between the dominance of these viruses, further emphasising the complex interplay between influenza and COVID-19 dynamics [34].

There were several models have been employed to predict pandemic trends for both influenza and COVID-19, ranging from short-term transmission dynamics models to long-term predictions using neural network models. As epidemic management normalises, the epidemiological scenario of co-transmission of these two viruses is increasingly recognised as a major trend [35-38]. Additionally, individuals are susceptible to co-infection with both influenza and SARS-CoV-2 pathogens at the same time [39,40], and studies have indicated that the case fatality rate for co-infected patients is twice that of those infected with SARS-CoV-2 alone [41]. Furthermore, this risk especially among the immunocompromised, fundamentally ill, and elderly populations, who are more vulnerable to severe outcomes [42,43]. The likelihood of co-transmission is influenced by factors such as the implementation of PHSMs and the level of population immunity. Also, we have observed that influenza vaccination, coupled with targeted interventions against SARS-CoV-2, could significantly reduce the incidence of influenza and the cumulative number of cases of cross-infection between influenza and SARS-CoV-2 in China [44]. Given these findings, relevant institutions must prepare in advance, promote seasonal influenza vaccination, plan for the allocation of scarce resources for prevention and control, and implement comprehensive measures to mitigate the impact of both influenza and SARS-CoV-2 on the population.

The average influenza incidence rates were (4.87 ± 2.20) per 10 000 for 2010–2019, (2.55 ± 1.25) per 10 000 for 2020–2022, and 17.00 per 10 000 for 2023. Before the emergence of COVID-19, from 2010 to 2019, the overall trend in influenza incidence rates was rising annually. However, during the period of COVID-19 and the implementation of PHSMs, the incidence rates were significantly lower than the previous trends. Similarly, before the COVID-19 pandemic, the mean β-value was (7.95 × 10^−10^) and the mean *R_t_*-value was (1.21 ± 0.16). During the COVID-19 & PHSMs period, both values decreased significantly to (3.17 × 10^−10^) for β and (0.86 ± 0.20) for *R_t_*, which were substantially lower than the lowest levels observed in the pre-COVID-19 and pre-PHSMs period. This reduction aligns with findings from other studies conducted during the same period [45,46].

Notably, in the post-COVID-19 and post-PHSMs period (2023), there has been a sudden and substantial increase in influenza incidence rates, and the β-value also returned to the fluctuation range observed from 2010–2019, while the *R_t_*-value increased to 2.25, indicating that on average, each infected person could transmit the virus to 2.25 susceptible individuals, suggesting rapid spread within the population. This resurgence is primarily attributed to high levels of population susceptibility maintained by the sustained implementation of PHSMs and the ongoing mutation and recombination of influenza pathogens, which may allow them to evade the human immune system. The co-existence of these factors led to a significant influenza outbreak following the complete termination of PHSMs. Studies have indicated that the magnitude of the influenza rebound correlates with the speed and extent of PHSMs termination [47].

Due to the differences in attitudes towards the COVID-19 epidemic among countries, each government’s PHSM response varied. A study showed that China, South Korea, and Singapore implemented strict preventive and control measures, so the epidemic can effectively be controlled. However, in the USA, the UK, and France, where mitigation policies were implemented, the prevention and control effects were insignificant, and the epidemic continued and even increased rapidly [48]. The strongest negative correlation (r = −0.678) observed between C8 (international travel controls) and influenza cases, which underscores the efficacy of restricting international travel in controlling the spread of influenza within China. In addition, measures H2 (testing policy), H1 (public information campaigns), and C2 (workplace closing) had significant impacts on the influenza epidemic. We can attribute the effectiveness of these measures to several factors:

1) early pathogen detection can rapidly identify infected persons and take relevant measures to control the further spread of influenza

2) enhancing public understanding and cooperation with epidemic prevention measures through information campaigns promotes the development of herd immunity

3) effectively reducing workplace population aggregation diminishes the potential for spreading the influenza virus.

These findings suggest that future epidemic prevention and control should emphasise a comprehensive approach, including early detection of pathogens, enhancing public awareness, and managing gatherings to contain outbreaks.

A comprehensive literature review indicates that, within the context of China, PHSMs represent the primary factor influencing influenza transmission, while other factors may have a limited impact [49,50]. A brief supplementary discussion of these factors is provided as follows:

1) reduced social contacts: daily interactions dropped by 7–8 times, with most limited to family settings, curbing the spread of both influenza and SARS-CoV-2 [51];

2) mask-wearing and hygiene: over 90% of the population adopted mask-wearing, and improved hygiene practices, particularly handwashing, further suppressed influenza transmission [52-55];

3) vaccination uptake: willingness to receive the influenza vaccine increased, with free vaccination programmes expected to enhance coverage [56];

4) health care system strain: resources diverted to COVID-19 management may reduce influenza detection and reporting [27].

Limited by the information and modelling constraints, this study still has some limitations. First, considering China's vast geographical diversity, varying climates, and the heterogeneous implementation of PHSMs across different regions, building models that account for variations across latitudes or administrative divisions could provide more representative insights. Second, the models used in this study did not stratify the population by essential characteristics such as gender and age, which may have simplified the structure. Lastly, due to the unavailability of complete influenza case data for 2024 and PHSM data only extending to the end of 2023, it is not feasible to fit models for influenza epidemics or evaluate transmission capacity for 2024, which also limits the understanding of the long-term impacts of PHSMs on influenza transmission post-pandemic.

Future directions for this research include the continuous collection of influenza incidence data and the ongoing adjustment and refinement of model parameters to enhance the accuracy of predictions concerning influenza activity post-COVID-19 and post-PHSMs. Such efforts will be crucial in adapting and improving public health strategies based on evolving epidemiological evidence.

## CONCLUSIONS

During the COVID-19 pandemic, we observed substantial fluctuations and changes in the intensity and scope of PHSMs implementation. Influenced by the emergence of SARS-CoV-2 and the implementation and subsequent termination of PHSMs, the dominant subtypes of influenza and the epidemic cycles in China exhibited significant variations. Throughout the COVID-19 and PHSMs period, the number of reported influenza cases, incidence rates, β-value, and *R_t_*-value significantly decreased, marking a departure from the trends observed from 2010 to 2019. However, in 2023, following the cessation of PHSMs, there was a complete rebound in these indicators. Preventive and control measures such as international travel controls, testing policies, public information campaigns, and workplace closures are scientifically validated and recommended for influenza transmission.

## Additional material


Online Supplementary Document
