# Supplementary material for: Quantitative assessment of Public Health and Social Measures implementation and relaxation on influenza transmission during COVID-19 in China: SEIABR and GBDT models
Source: J Glob Health. 2024 Dec 27;14:05038. doi: 10.7189/jogh.14.05038 (PMC11672222; doi:10.7189/jogh.14.05038)
Supplement: Online Supplementary Document [file jogh-14-05038-s001.pdf]

Appendix materials

Table of epidemic prevention and control policy indicators

PHSMs	Degree	Range	Intensity
C1	(0) No measures	0	0
School closing	(1) Recommend closing, or all schools open with	1	17
	alterations resulting in significant differences	2	33
	compared to usual, non-Covid-19 operations		
	(2) Require closing (only for some levels or	3	50
	categories, eg just high schools, or just public	4	67
	schools)		
	(3) Require closing all levels	5	83
		6	100
C2	(0) Not closed	0	0
Workplace	(1) Recommend closing (or work from home)	1	17
closing		2	33
	(2) Require closing (or work from home) for some	3	50
	sectors or categories of workers	4	67
	(3) Require closing (or work from home) for all-	5	83
	but-essential workplaces (e.g. grocery stores,	6	100
	doctors)		
C3	(0) Not cancelled	0	0
Cancel public	(1) Recommended cancellation	1	25
events		2	50
	(2) Demand cancellation	3	75
		4	100
C4	(0) No restrictions	0	0
Restrictions on	(1) Restrictions on very large gatherings (the limit	1	12.5
gatherings	is above 1000 people)	2	25
	(2) Restrictions on gatherings between 101-1000	3	37.5
	people	4	50
	(3) Restrictions on gatherings between 11-100	5	62.5
	people	6	75
	(4) Restrictions on gatherings of 10 people or less	7	87.5
		8	100
C5	(0) Not closed	0	0
Close public	(1) Recommended closure or reduction of	1	25
transport	operations	2	50
	(2) Request to close all	3	75
		4	100
C6	(0) No requirement	0	0
Stay at home	(1) Recommended home quarantine	1	17
requirements		2	33
	(2) Request not to go out unless necessary	3	50

		4	67
	(3) Request to home quarantine	5	83
		6	100
C7	(0) No limit	0	0
Restrictions on	(1) Recommended limitation	1	25
internal		2	50
movement	(2) Mandatory restriction	3	75
		4	100
C8	(0) No requirement	0	0
International	(1) Nucleic acid testing of personnel from high-	1	17
travel controls	risk sources	2	33
	(2) Personnel from high-risk sources are	3	50
	prohibited from entering	4	67
	(3) Close the country's borders	5	83
		6	100
H1	(0) No information campaigns	0	0
Public	(1) Recommended campaigns	1	25
information		2	50
campaigns	(2) Demand campaigns	3	75
		4	100
H2	(0) No testing policy	0	0
Testing policy	(1) Test a subset of people with symptoms	1	17
		2	33
	(2) Test the entire population with symptoms	3	50
		4	67
	(3) Nucleic acid testing for all personnel	5	83
		6	100
H3	(0) No contact tracing	0	0
Contact tracing	(1) Finite traceability	1	25
		2	50
	(2) Comprehensive traceability	3	75
		4	100
H6	(0) No policy	0	0
Facial	(1) Recommended to wear a mask in public	1	12.5
Coverings		2	25
	(2) Required in certain public places (hospitals, banks, etc.)	3	37.5
		4	50
	(3) required t in all public places	5	62.5
		6	75
	(4) Required in all public places and at home	7	87.5
		8	100
